# Causative Microorganisms of Infectious Endophthalmitis: A 5-Year Retrospective Study

**DOI:** 10.1155/2016/6764192

**Published:** 2016-06-19

**Authors:** Fang Duan, Kaili Wu, Jingyu Liao, Yongxin Zheng, Zhaohui Yuan, Junlian Tan, Xiaofeng Lin

**Affiliations:** Zhongshan Ophthalmic Center, State Key Laboratory of Ophthalmology, Key Laboratory of Ophthalmology and Visual Science, Sun Yat-sen University, 54 Xianlie Road, Guangzhou, Guangdong 510060, China

## Abstract

This study aimed to identify the microbial etiology of infectious endophthalmitis and to determine the antibacterial susceptibilities of bacterial isolates at an eye hospital in South China. A retrospective analysis was carried out on 330 patients with clinically diagnosed infectious endophthalmitis who underwent microbiological evaluation from January 2010 to December 2014. Of the 330 patients, 193 patients (58.5%) had posttraumatic endophthalmitis, 67 patients (20.3%) had postoperative endophthalmitis, 61 patients (18.5%) had endogenous endophthalmitis, and 9 patients (2.7%) had postcorneal infective endophthalmitis. Of the 105 cases (31.8%) of culture-positive endophthalmitis, 79 cases (75.2%) had bacterial growth and 26 cases (24.8%) had fungal growth. In posttraumatic endophthalmitis, Gram-positive bacteria were the predominant species, followed by Gram-negative bacteria and fungi. In endogenous endophthalmitis, Gram-negative bacteria were the predominant species, followed by fungi and Gram-positive bacteria. In postsurgical endophthalmitis, all infections were bacterial. However, in postcorneal infective endophthalmitis, all infections were fungal. Overall, levofloxacin showed the highest activity against bacterial isolates. There was a significant difference in the susceptibility to tobramycin between the isolates from posttraumatic and postoperative endophthalmitis (*p* < 0.05). The results of this study identify the microbial spectrum of infectious endophthalmitis in this clinical setting.

## 1. Introduction

Infectious endophthalmitis, a potentially sight-threatening disease, is characterized by marked inflammation of intraocular tissues and fluids. The causative pathogen of endophthalmitis can come from the outside environment or from systemic infections transported in the bloodstream. Infectious endophthalmitis can be divided into the broad categories exogenous and endogenous. Exogenous endophthalmitis is caused by inoculation of the eye by microorganisms from the external environment and most commonly occurs as a complication of ocular surgery or trauma. Occasionally, it results from the contagious spread of infectious microbes from the cornea. Endogenous endophthalmitis is caused by hematogenous spread of infectious organisms from distant sites in the body. Both categories of endophthalmitis lead to subsequent intraocular inflammation and potentially severe visual loss.

Posttraumatic endophthalmitis is an important complication of open globe injury, and the incidence has been reported in recent years to vary widely from 0.9% to 11.91% [1–5]. The spectrum of causative organisms varies and depends on the region and environment, the type of injury, the living environment, and the time from injury to wound repair [6–8]. Bacteria account for approximately 80%–90% of culture-positive cases [9, 10], and Gram-positive cocci are the most common isolates among these bacteria, followed by Gram-positive bacilli and other Gram-negative organisms. Postoperative endophthalmitis can occur after any intraocular procedure, such as cataract surgeries [11], pars plana vitrectomy [12], penetrating keratoplasty [13], scleral buckling with drainage of subretinal fluid [14], bleb-related infections after trabeculectomy [15], or implantation of a glaucoma drainage device [16]. More recently, cases of postinjection endophthalmitis have occurred due to the use of intravitreal injection of vascular endothelial growth factor antagonists [17]. The organisms recovered from postoperative endophthalmitis usually originate from the conjunctiva, eyelid, or nose of the patient [18, 19]. The most commonly identified organisms are Gram-positive bacteria [20, 21]. In contrast to exogenous endophthalmitis, endogenous endophthalmitis results from the hematogenous spread of microorganisms from distant foci and across the blood-ocular barrier [22]. According to the previous studies of Bhoomibunchoo et al. [23] and Fan et al. [24], endogenous endophthalmitis accounted for 3.3% and 16%, respectively, of all reported endophthalmitis cases. Both bacterial and fungal agents are noted as potential causative agents of endogenous endophthalmitis; fungal organisms account for the majority of cases [25, 26]. However, in Asian studies, bacteria are predominant causes to endogenous endophthalmitis [27, 28].

Most reports of endophthalmitis have focused mainly on a certain type of endophthalmitis. To better understand the specific microbial pathogens responsible for the development of the various forms of endophthalmitis in South China, the present study retrospectively investigated and compared the spectrum of microbial pathogens that caused postoperative, posttraumatic, and postcorneal infective and endogenous endophthalmitis. In addition, the in vitro susceptibility of bacterial isolates from each type of endophthalmitis to eight antibiotics was assessed. These findings will help in the implications for clinical treatment.

## 2. Materials and Methods

A retrospective review was conducted on inpatients who were diagnosed with or suspected to have infectious endophthalmitis at the Zhongshan Ophthalmic Center, Guangzhou, from January 2010 to December 2014. This study was performed in compliance with the principles of the Declaration of Helsinki and was approved by the Institutional Ethics Committee of Zhongshan Ophthalmic Center, Sun Yat-sen University. According to the possible sources of infectious endophthalmitis, the patients were divided into four groups as follows: endophthalmitis with trauma, endophthalmitis associated with microbial keratitis, endogenous endophthalmitis, and endophthalmitis with postoperative infection after sterile ocular surgery, including cataract surgeries, vitrectomy, penetrating keratoplasty, scleral buckling, trabeculectomy, implantation of a glaucoma drainage device, and intravitreal injection.

### 2.1. Pathogen Isolation and Identification

Samples were taken from diseased tissues from all patients with suspected or diagnosed infectious endophthalmitis. In detail, the samples of endophthalmitis due to corneal ulcer were obtained from the cornea after topical anesthesia using 0.5% proparacaine hydrochloride, whereas other samples of endophthalmitis were taken from the aqueous humor and/or vitreous fluid during surgery. All protocols were conducted according to our established methods [29]. Briefly, cornea specimens were sampled by scraping the base and edges of the ulcerated part of the cornea with a sterile special knife. Fluids from the anterior chamber were aspirated through the limbus using a needle on a 1 mL syringe. Vitreous specimens were obtained through the pars plana prior to antibiotic injection or vitrectomy.

The collected samples were then inoculated in nutrient broth overnight at 37°C. Subsequently, the broth was inoculated onto sheep blood agar and potato glucose agar for the growth of bacterial cultures and fungal cultures, respectively. Bacteria isolates were identified using an automated microbiological system (Vitek 2 Compact, BioMerieux, Inc., Durham, NC, USA); fungi isolates were identified by technicians with working experience according to the morphology of fungi.

### 2.2. Antibiotic Susceptibility Test

Antibiotic susceptibility testing of isolated bacteria was performed using the traditional disc diffusion method. The antibiotic susceptibility was determined in accordance with the methods of the Clinical and Laboratory Standards Institute (CLSI). Bacterial susceptibilities were recorded as “resistant,” “intermediate,” or “sensitive.” For the purpose of this study, being “intermediate” and being “sensitive” were both considered sensitive.

### 2.3. Statistical Analysis

The statistical analysis was performed using SPSS 17.0 software (Chicago, IL, USA). The chi-square test was used to compare categorical variables. Differences were considered to be significant at *p* < 0.05.

## 3. Results

**Figure 1:**
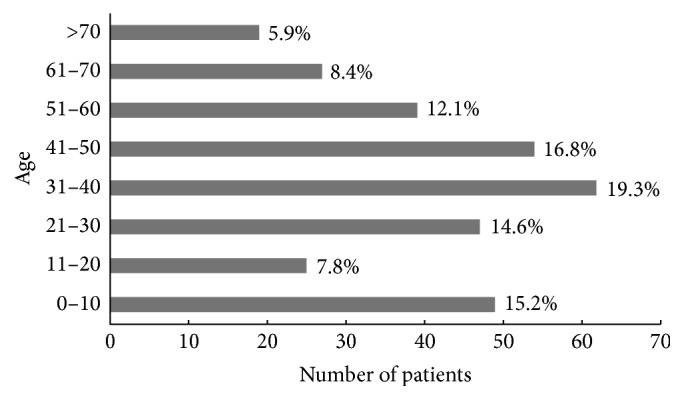
Demographics of the 330 patients with infectious endophthalmitis. Among the patients in the 11- to 20-year age group, 5.2% of these patients were aged 11–17 years.

During the 5-year study period, samples from 330 patients (242 men, 88 women) who presented with endophthalmitis were collected and subjected to microbiological analysis (mean age ± SD, 37.3 ± 20.8 years; range, 1–87 years). The demographic analysis of the patients is shown in Figure 1. Patients with ages of 31–40 years and 41–50 years constituted 19.3% and 16.8%, respectively, of all patients. Patients younger than 11 years were 15.2% of all patients, followed by patients with ages of 21–30 years and 51–60 years. The patients of working age constituted 62.8% of all patients. Of the 330 patients, 193 patients (58.5%) presented with endophthalmitis after open globe injuries, 67 patients (20.3%) presented with endophthalmitis after intraocular surgery, 61 patients (18.5%) presented with endogenous endophthalmitis, and 9 patients (2.7%) presented with postcorneal infective endophthalmitis. In detail, of 67 patients with postoperative endophthalmitis, 42 occurred after cataract surgery, 15 occurred after glaucoma bleb surgery, 9 occurred after vitrectomy, and 1 occurred after intravitreal injection of anti-VEGF agents (Lucentis). As for the 15 bleb-related endophthalmitis, only one patient occurred at seventh day after operation; the rest of them occurred more than 30 days after operation, and onset time ranged from 4 months to 15 years after first glaucoma bleb surgery. In the 67 patients with ages of 0–17 years, 54 patients (80.6%) presented with endophthalmitis after open globe injuries, 2 patients (3%) presented with endophthalmitis after intraocular surgery, 10 patients (14.9%) presented with endogenous endophthalmitis, and 1 patient (1.5%) presented with postcorneal infective endophthalmitis. Bacterial culture was positive in 13 patients and fungi culture was positive in 8 patients, a significantly higher rate of posttraumatic endophthalmitis in patients with ages of 0–17 years than in patients aged 18 or older (80.6% versus 52.9%, *p* < 0.01).

**Figure 2:**
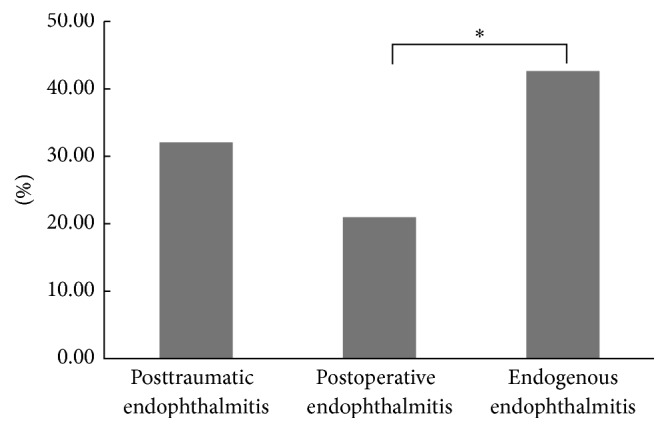
The positive rates of the microorganism cultures. ^*∗*^ *p* < 0.05.

The overall culture-positive rate of samples was 31.8% (105/330). Among the culture-positive samples, 79 samples (75.2%) were from exogenous endophthalmitis, and 26 samples (24.8%) were from endogenous endophthalmitis. The culture-positive rates of samples from patients with posttraumatic, postoperative, or endogenous endophthalmitis were assessed (Figure 2). The highest culture-positive rate was found in endogenous endophthalmitis (42.6%; 26 of 61), followed by posttraumatic endophthalmitis (32.1%; 62 of 193) and postoperative endophthalmitis (20.9%; 14 of 67). In addition, there was a significant difference between the culture-positive rates of endogenous endophthalmitis and those of postoperative endophthalmitis (*p* < 0.05). Of the 61 patients with clinically diagnosed endogenous endophthalmitis, 26 had positive intraocular cultures. Blood cultures were obtained on only 9 of these 61 patients and 2 were positive. One of the positive blood cultures also had positive intraocular cultures; the other had positive blood cultures but negative intraocular cultures. Because positive samples were found in only 3 patients with postcorneal infective endophthalmitis, this culture-positive rate was not compared to those of other groups.

The microbial pathogens recovered from eyes with infectious endophthalmitis are shown in Table 1. Of these isolates, Gram-positive bacteria were found to be the predominant cause of endophthalmitis (45.7%, 48 of 105), followed by Gram-negative bacteria (29.5%, 31 of 105) and fungi (24.8%, 26 of 105). Specifically, the main causative organisms for infections were all* Staphylococcus* spp. (31.4%, 33 of 105); among them,* Staphylococcus* epidermidis (15.2%, 16 of 105) was the predominant isolate. In addition, two cases were found with a mixed infection of Gram-negative bacteria and fungi. Of 67 patients with postoperative endophthalmitis, 14 were culture-positive. In detail, postcataract cases were due to coagulase-negative staphylococci in 3,* Enterococcus* in 2,* Pseudomonas aeruginosa* in 1,* Serratia marcescens* in 1, and* Xanthomonas* in 1. Postvitrectomy cases were due to coagulase-negative staphylococci in 3 and* Enterococcus* in 1. Bleb-related cases were due to* Klebsiella* in 1 and* Acinetobacter junii* in 1.

**Figure 3:**
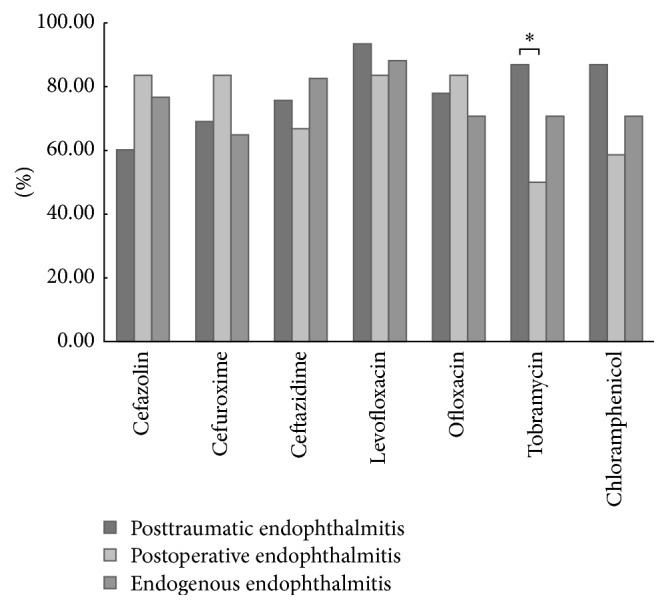
The susceptibilities of bacterial isolates from infectious endophthalmitis to seven different antibiotics. ^*∗*^ *p* < 0.05.

The antibiotic susceptibilities were compared for bacteria isolated from samples from patients with posttraumatic, postoperative, or endogenous endophthalmitis for the following antibiotics: ceftazidime, cefuroxime, cefazolin, levofloxacin, ofloxacin, tobramycin, and chloramphenicol (Figure 3). In detail, the bacteria in posttraumatic endophthalmitis samples were more susceptible to levofloxacin, followed by tobramycin and chloramphenicol. The bacteria in the postoperative endophthalmitis samples were more susceptible to cefazolin, cefuroxime, levofloxacin, and ofloxacin. The bacteria in endogenous endophthalmitis samples were most susceptible to levofloxacin, followed by ceftazidime. There was a significant difference in the susceptibility to tobramycin between bacterial isolates from posttraumatic and postoperative endophthalmitis samples (*p* < 0.05).

## 4. Discussion

In our study of 330 patients with infectious endophthalmitis, most cases were associated with ocular trauma. This finding is dissimilar to previous reports in which most cases developed endophthalmitis after intraocular surgery in the United States [30], the United Kingdom [31], and Australia [32]. However, Bhoomibunchoo et al. [23] reported that posttraumatic endophthalmitis constituted the majority of infectious endophthalmitis cases in northeastern Thailand, which is consistent with our results. The discrepancy in results might be attributable to the higher incidence of ocular injury that occurs with industrial and agriculture procedures in developing countries. We found that endophthalmitis occurred predominantly in men (73.3%), in accordance with other studies. This finding might be due to gender-based behavior and male involvement in higher risk working activities.

The isolation of these causative organisms is an important step in clinical practice. Overall, our study revealed that the major causative pathogens for infectious endophthalmitis were Gram-positive organisms, followed by Gram-negative organisms and fungi, which is consistent with reports of previous studies [23, 33]. A total of 105 (31.8%) culture-positive isolates were identified from 330 cases of infective endophthalmitis over 5 years. The culture-positive rates were close to those reported from previous studies [9, 23]. In comparing the culture-positive samples in different groups, we found that the highest culture-positive rate was in endogenous endophthalmitis (42.6%; 26 of 61), followed by posttraumatic endophthalmitis (32.1%; 62 of 193) and postoperative endophthalmitis (20.9%; 14 of 67). In addition, there was a significant difference between endogenous endophthalmitis and postoperative endophthalmitis (*p* < 0.05). A study in South India [33] reported that the highest rate of culture positivity was in specimens from eyes with endophthalmitis due to trauma (65%; 141 of 217), followed by postsurgical endophthalmitis (36%; 225 of 625) and endogenous endophthalmitis (15.5%; 11 of 71); these findings are dissimilar to our results. Many factors may have contributed to this discrepancy because the spectrum and virulence of causative organisms varied depending on the region and the environment. On the other hand, the patients had been treated for infections before they were referred to tertiary care, particularly the patients with ocular trauma or perioperative endophthalmitis.

In the group of patients with endophthalmitis following open globe injuries, Gram-positive isolates were found in 32 eyes (51.6%), Gram-negative isolates were found in 16 eyes (25.8%), and fungal isolates were found in 14 eyes (22.6%).* Staphylococcus* spp. were the major causative pathogens, which is consistent with previous reports of posttraumatic endophthalmitis [7–9]. Posttraumatic endophthalmitis caused by a fungus is less common than that caused by bacteria. In our study,* Aspergillus* spp. was the most common fungal species, which is consistent with previous reports [33–35], followed by* Fusarium* spp. and* Candida* spp. For postoperative endophthalmitis, the Gram-positive isolates occurred in 9 eyes (64.3%), and Gram-negative isolates occurred in 5 eyes (35.7%); no fungal isolates occurred. Endogenous endophthalmitis occurs when organisms reach the eye through the bloodstream. A review of the literature shows that endogenous endophthalmitis constitutes 2%–17% of the cases of infectious endophthalmitis [36]. However, Krause et al. [37] reported a prevalence as high as 41%. In our study, clinically diagnosed endogenous endophthalmitis comprised 18.5% (61 of 330) of the cases of infectious endophthalmitis. In detail, in endogenous endophthalmitis the Gram-positive isolates occurred in 7 eyes (26.9%), Gram-negative isolates occurred in 10 eyes (38.5%), and fungal isolates occurred in 9 eyes (34.6%). The proportion of fungal infections was much lower than in Western nations and Australia. For example, Connell's study had 41 culture-positive endogenous endophthalmitis cases, and of these 27 were fungi but 21 of these fungal cases were due to* Candida* [26]. Similarly, Leibovitch reported results for 13 patients, of whom 8 had fungal isolates and 6 of these 8 were due to* Candida* [38]. Schiedler et al. reported results for 21 culture-positive endogenous endophthalmitis cases, and 13 were due to fungi but 7 of these 13 were due to* Candida* [25]. In our study, 26 culture-positive endogenous endophthalmitis cases, 9 were due to fungi. In detail, 5 molds, 2* Candida*, and 2* Fusarium* were identified. We found a lower percentage of* Candida* in endogenous fungal endophthalmitis cases than the Australian and Florida studies. In studies in Asia, fungi are the causative organisms in approximately 17.5% to 28.9% of total cases of endogenous endophthalmitis, whereas the rest of the cases are attributed to bacterial causes [27, 28]; these findings are similar to our results. In addition, of the 9 patients who presented with endophthalmitis due to infective keratitis, 3 patients were identified with fungal infections while no bacteria were isolated. This finding might be because fungi are more difficult to control than bacteria.

Bacterial susceptibilities to 7 antibiotics were tested in our study. Overall, levofloxacin showed the highest activity against bacterial isolates, which was consistent with a previous study [29]. In detail, the bacteria in posttraumatic endophthalmitis samples were more susceptible to levofloxacin, followed by tobramycin and chloramphenicol. The bacteria in postoperative endophthalmitis samples were more susceptible to cefazolin, cefuroxime, levofloxacin, and ofloxacin. However, the susceptibility to tobramycin was only 50%. The bacteria in endogenous endophthalmitis samples were most susceptible to levofloxacin, followed by ceftazidime and cefazolin. There was a significant difference in susceptibility to tobramycin between the isolates from posttraumatic and postoperative endophthalmitis samples (*p* < 0.05). Analysis of the in vitro susceptibility patterns showed variations in the susceptibility of isolates from different clinical settings. Our present sensitivity data for antibiotics will help clinician in choice of antibiotics for endophthalmitis before definitive information on the causative pathogenic microorganisms is available.

In conclusion, we analyzed the microbial etiology of infectious endophthalmitis in 330 patients. Of these, 193 patients (58.5%) had posttraumatic endophthalmitis, 67 patients (20.3%) had postoperative endophthalmitis, 61 patients (18.5%) had exogenous endophthalmitis, and 9 patients (2.7%) had postcorneal infective endophthalmitis. Of 105 cases (31.8%) of culture-positive endophthalmitis, 79 cases (75.2%) had bacterial growth, and 26 cases (24.8%) had fungal growth. In addition, a high percentage of bacteria were primarily susceptible to levofloxacin.

## Figures and Tables

**Table 1:** Microorganisms isolated and cultured from samples from endophthalmitis patients.

	Posttraumatic (*N* = 62)	Postoperative (*N* = 14)	Endogenous (*N* = 26)	Corneal infective (*N* = 3)	Total (number %)
*Gram-positive organisms*	*32*	*9*	*7*	*0*	*48 (45.7)*
*Staphylococcus* spp.^*∗*^	22	6	5	0	33 (31.4)
*Kocuria* spp.	1	0	0	0	1 (1.0)
*Streptococcus* spp.	2	0	1	0	3 (2.9)
*Micrococcus* spp.	2	0	0	0	2 (1.9)
*Bacillus* spp.	2	0	0	0	2 (1.9)
*Enterococcus* spp.	0	3	1	0	4 (3.8)
*Corynebacterium* spp.	1	0	0	0	1 (1.0)
Other	2	0	0	0	2 (1.9)
*Gram-negative organisms *	*16*	*5*	*10*	*0*	*31 (29.5)*
*Pseudomonas* spp.	1	1	3	0	5 (4.8)
*Burkholderia* spp.	1	0	0	0	1 (1.0)
*Enterobacter* spp.	3	0	1	0	4 (3.8)
*Serratia* spp.	1	1	0	0	2 (1.9)
*Chryseobacterium* spp.	1	0	0	0	1 (1.0)
*Xanthomonas* spp.	1	1	2	0	4 (3.8)
*Sphingomonas* spp.	1	0	0	0	1 (1.0)
*Shigella* spp.	1	0	0	0	1 (1.0)
*Providencia* spp.	2	0	0	0	2 (1.9)
*Aeromonas* spp.	1	0	1	0	2 (1.9)
*Klebsiella* spp.	0	1	3	0	4 (3.8)
Other	3	1	0	0	4 (3.8)
*Fungi*	*14*	*0*	*9*	*3*	*26 (24.8)*
*Aspergillus* spp.	7	0	1	1	9 (8.6)
*Fusarium* spp.	3	0	4	1	8 (7.6)
*Candida* spp.	1	0	2	0	3 (2.9)
Other^#^	3	0	2	1	6 (5.7)

^*∗*^Of 33 *Staphylococcus* spp. isolates, 17 were coagulase-negative staphylococci. Ten isolates were in posttraumatic endophthalmitis group, and 5 and 2 isolates were in postoperative and endogenous endophthalmitis group, respectively.

^**#**^Other fungi included *Bipolaris sorokiniana*, *Mucor*, and *Penicillium*.
